# Chronic Lower Lip Swelling due to Granulomatous Cheilitis

**DOI:** 10.1002/ccr3.72669

**Published:** 2026-05-06

**Authors:** Jesus Ruiz, Jacob Garner

**Affiliations:** ^1^ Department of Family Medicine University of North Carolina at Chapel Hill School of Medicine Chapel Hill North Carolina USA

**Keywords:** dermatology, granulomatous cheilitis, lip swelling, orofacial edema

## Abstract

Granulomatous cheilitis should be considered in the differential diagnosis of persistent, unexplained orofacial swelling, particularly when standard treatments fail.

## 1 Case Presentation

A 58‐year‐old female presented to her primary care office to re‐establish care and was noted to have persistent lower lip swelling for 3 years without resolution. She had previously been treated with oral amoxicillin–clavulanate (875 mg twice daily for 10 days) and a short course of oral prednisone (40 mg daily for 5 days) without improvement. She had been taking lisinopril for hypertension for 5 years prior to symptom onset. Given concern for angioedema, the medication was discontinued; however, there was no improvement after 1 year off therapy.

The patient denied facial weakness, tongue changes, gastrointestinal symptoms, or systemic complaints such as fever or weight loss.

**FIGURE 1:**
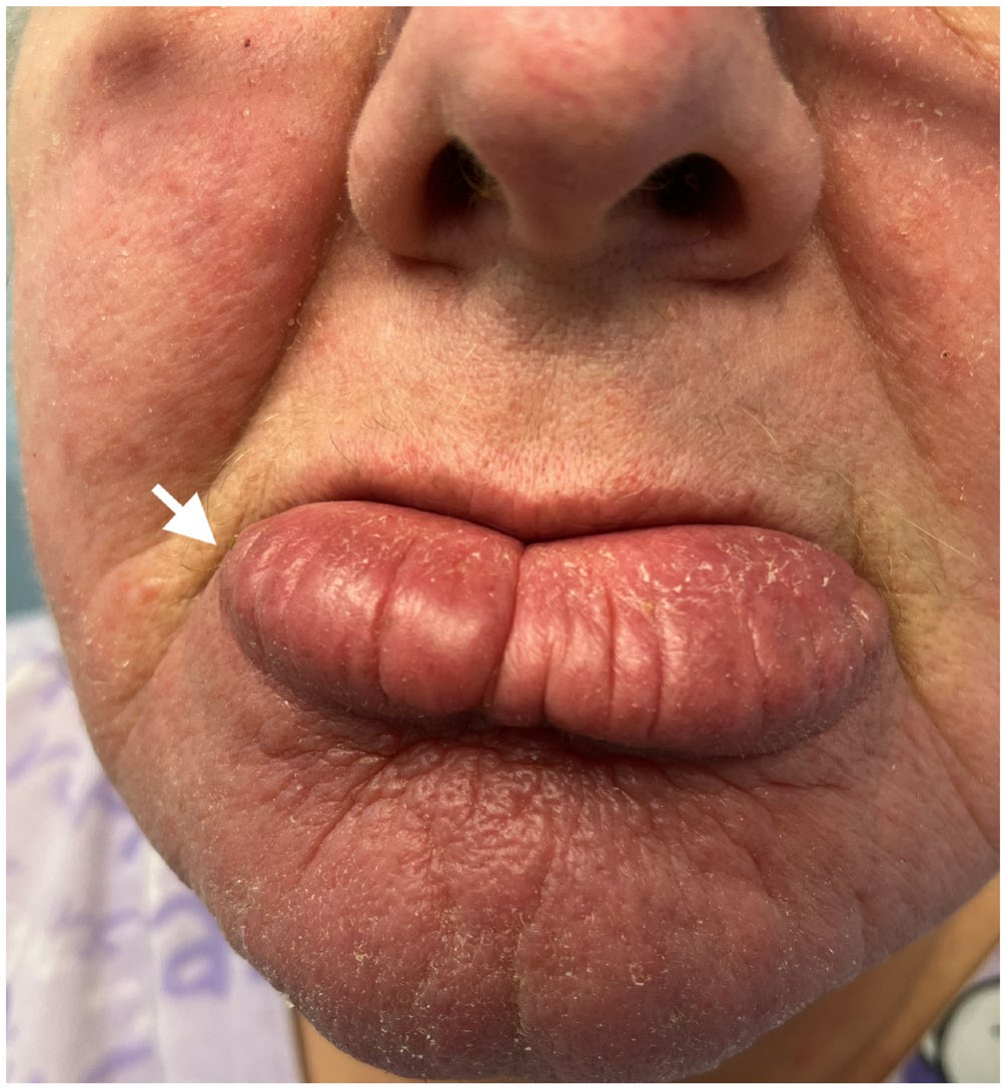
Chronic lower lip and chin swelling causing hypertrophy, induration, and erythema.

On examination, she was well appearing with stable vital signs. There was significant erythematous, non‐tender, and indurated swelling of the lower lip extending to the chin (Figure 1). Intraoral examination showed no gingival hypertrophy, mucosal ulceration, or cobblestone appearance of the buccal mucosa. The tongue was normal without fissuring, and no intraoral lesions were identified. An elliptical incisional biopsy of the lower lip was performed. Histopathology demonstrated noncaseating granulomatous inflammation composed of epithelioid histiocytes and multinucleated giant cells, with associated lymphocytic infiltrate and no necrosis.

## 2 Clinical Image Question

What is the most likely diagnosis in a patient with chronic, non‐tender, indurated lower lip swelling with biopsy demonstrating noncaseating granulomatous inflammation, unresponsive to antibiotics and corticosteroids?

**Answer:** Granulomatous cheilitis (GC).

## 3 Discussion and Outcome

GC is a rare condition of unclear etiology, primarily affecting adults, characterized by chronic, painless lip swelling and histologic non‐necrotizing granulomas with associated lymphedema, inflammation, and fibrosis. When accompanied by facial palsy and lingua plicata, it is termed Melkersson‐Rosenthal syndrome. Diagnosis is clinical but requires biopsy confirmation [1, 2]. The differential includes cellulitis, angioedema, rosacea, and other granulomatous diseases.

Distinguishing GC from Crohn's disease, sarcoidosis, and other systemic granulomatous conditions is essential. Recommended evaluation includes complete blood count, erythrocyte sedimentation rate, C‐reactive protein, chest imaging, serum angiotensin‐converting enzyme levels, and tuberculosis screening. Endoscopy may be considered if gastrointestinal symptoms are present. In this case, laboratory evaluation (CBC, ESR, CRP, QuantiFERON gold) was unremarkable. Serum angiotensin‐converting enzyme levels were normal, and chest imaging showed no evidence of sarcoidosis. The patient had no gastrointestinal symptoms suggestive of Crohn's disease.

There is no standardized treatment for GC. Management is variable, with commonly used therapies including intralesional corticosteroids, systemic immunosuppressants, antibiotics (e.g., tetracyclines), and dietary modification (avoidance of cinnamon and benzoates). Surgical reduction has also been described [3].

The patient was treated with oral doxycycline (100 mg twice daily for 14 days), a short course of oral prednisone (40 mg daily for 5 days), and three serial intralesional triamcinolone acetonide injections (20 mg/mL) administered every 4 weeks. She was also counseled on avoidance of cinnamon‐ and benzoate‐containing foods. This resulted in significant improvement in lip swelling and erythema, although mild residual swelling persisted at the most recent follow‐up. The patient was subsequently lost to follow‐up before additional intralesional corticosteroid therapy could be completed.

## Author Contributions

**Jesus Ruiz:** conceptualization, data curation, formal analysis, writing – original draft, writing – review and editing. **Jacob Garner:** conceptualization, investigation, writing – original draft, writing – review and editing.

## Funding

The authors have nothing to report.

## Consent

The patient gave written informed consent to publish this report in accordance with the journal's patient consent policy.

## Conflicts of Interest

The authors declare no conflicts of interest.

## Data Availability

The data that support the findings of this study are available from the corresponding author upon reasonable request.
